# Golgi retention of KIT in gastrointestinal stromal tumour cells is phospholipase D activity-dependent

**DOI:** 10.1038/s41598-025-14739-w

**Published:** 2025-08-06

**Authors:** Yuuki Obata, Miyuki Natsume, Isamu Shiina, Tsuyoshi Takahashi, Toshirou Nishida

**Affiliations:** 1 Laboratory of Intracellular Traffic & Oncology, National Cancer Center Research Institute, Tsukiji, 5-1-1, Chuo-ku, Tokyo, 104-0045 Japan; 2 Department of Applied Chemistry, Faculty of Science, Tokyo University of Science, Shinjuku-ku, Tokyo, 162-8601 Japan; 3 Department of Gastroenterological Surgery, Graduate School of Medicine, Osaka University, Suita, 565-0871 Osaka Japan; 4 National Cancer Center Hospital, Tsukiji, Chuo-ku, Tokyo, 104-0045 Japan

**Keywords:** RTK, KIT, GIST, Golgi/TGN, PKD, PLD, Biochemistry, Cancer, Cell biology

## Abstract

A constitutively active mutant of the receptor protein tyrosine kinase KIT is a major cause of gastrointestinal stromal tumours (GISTs). Recently, we discovered that, during biosynthetic transport, the KIT mutant (KIT^mut^) is retained in the Golgi/*trans-*Golgi network (TGN), where it activates downstream molecules. This retention is dependent on the phospholipase Cγ2–protein kinase D2–PI4 kinase IIIβ (PLCγ2–PKD2–PI4KIIIβ) pathway, which KIT^mut^ activates at the Golgi/TGN. The activated cascade aberrantly recruits GGA1 and the γ-adaptin subunit of AP1, resulting in KIT^mut^ retention in the Golgi/TGN. However, the precise mechanisms, including the mediators and effectors of the pathway, remain unclear. In humans, the phosphatidic acid-generating enzymes, phospholipase D1 (PLD1) and PLD2 are known downstream proteins of PKD. In the presence of the PLD inhibitor CAY10594, KIT^mut^ is released from the Golgi/TGN and subsequently degraded in lysosomes, leading to signal inactivation. Knockdown experiments indicated that PLD2 plays a role in KIT^mut^ retention. KIT^mut^ activates PLD2 through PKD2, but not PI4KIIIβ, for Golgi/TGN retention. PLD activity is required for the association of γ-adaptin with GGA1. Therefore, the KIT–PLCγ2–PKD2 pathway separately activates PLD2 and PI4KIIIβ to recruit γ-adaptin and GGA1. Collectively, these results suggest that KIT^mut^ retention is dependent on the activation of the PLCγ2–PKD2–PLD2 cascade in GIST cells.

**Supplementary Information:**

The online version contains supplementary material available at 10.1038/s41598-025-14739-w.

## Introduction

The stem cell factor (SCF) receptor KIT is a type III receptor protein tyrosine kinase (RTK) primarily located in the plasma membrane (PM) of the interstitial cells of Cajal in the gastrointestinal tract, as well as in haematopoietic cells, mast cells, melanocytes, and germ cells in the testis^1–3^. Upon binding of SCF to the PM, KIT dimerises, resulting in enhanced protein tyrosine kinase activity. This, in turn, activates several signalling molecules, such as AKT, extracellular signal-regulated kinase, phospholipase Cγ (PLCγ), and signal transducer and activator of transcription (STAT) proteins, leading to cell growth, survival, migration, and differentiation^1^. Consequently, the constitutive activation of KIT through genetic abnormalities such as point mutations and in-frame deletions plays a critical role in cancerogenesis. Indeed, most cases of mast cell leukaemia in adult humans, as well as patients with gastrointestinal stromal tumours (GISTs), harbour KIT mutations^4–7^. As a result, imatinib, a KIT inhibitor (tyrosine kinase inhibitor (TKI)), has been used to treat patients with advanced GISTs. However, 2–3 years after imatinib administration, TKIs become ineffective in treating advanced GISTs^8–10^. In most cases, mutations conferring imatinib resistance are found in the *KIT* gene.

Previously, we reported that, unlike normal KIT, mutant KIT (KIT^mut^) in leukaemia cells and GIST is localised to intracellular compartments where it can be autophosphorylated and activated^11–15^. Moreover, the localisation of the mutant in leukaemia cells is notably different from that in GIST cells; in leukaemia cells, KIT^mut^ accumulates in the endosome–lysosome compartments^11,13,15^whereas in GIST cells, it is retained in the Golgi/*trans*-Golgi network (Golgi/TGN) area during biosynthesis^12,14^. In both cases, KIT^mut^ localises to intracellular compartments depending on its tyrosine kinase activity^11,12,15^. We recently demonstrated that KIT^mut^ retention depends on protein kinase D2 (PKD2) activity in GIST cells^16^. The mediator between KIT^mut^ and PKD2 is PLCγ2. It was suggested that the KIT–PLCγ2–PKD2 pathway activates the phosphatidylinositol 4-phosphate-producing (PI4P-producing) enzyme PI4 kinase IIIβ (PI4KIIIβ), leading to the aberrant recruitment of Golgi-localised, γ-ear-containing, ARF-binding family of protein 1 (GGA1) and an adaptor protein-1 complex (AP1) component γ-adaptin. Subsequently, KIT itself is retained in the Golgi/TGN area. Although previous studies showed that GGA1 and AP1 carry cargoes from the TGN^17^we assume that aberrantly, probably excessively, recruited adaptor proteins disrupt the normal trafficking of KIT. These findings raised several questions: How do aberrantly recruited AP1 and GGA1 trap KIT in the Golgi/TGN?; Are there PKD2 effectors other than PI4KIIIβ? However, these questions remain unanswered.

PKD enhances phosphatidylcholine-selective phospholipase D (PC-PLD; hereafter referred to as PLD) activity to generate phosphatidic acid (PA) from PC on the cytoplasmic face of the PM and Golgi/TGN membrane^18–20^. Under normal conditions, PA plays a key role in the formation of transport carriers in the Golgi/TGN membrane^21–26^. It creates a negative curvature at the budding site of membrane carriers owing to its cone-like shape and provides a hydrophobic zone as a scaffold region for functional proteins^27–29^. Human PLDs comprise six structurally related proteins: PLD1, PLD2, PLD3, PLD4, PLD5, and PLD6. PLD1 and PLD2 are crucial for membrane carrier formation through PLD activity, whereas PLD3, PLD4, and PLD5 lack canonical phospholipase activity and may possess endonuclease activity in lysosomes and the endoplasmic reticulum (ER)^29,30^. PLD6 is localised in mitochondria to produce PA from cardiolipins. Therefore, we hypothesised that the KIT–PLCγ2–PKD2 pathway activates PLD1 and/or PLD2, and that aberrant PA production leads to the mutant receptor’s retention in the Golgi/TGN in GIST cells.

The present study aimed to determine the requirement of PLD activity for Golgi/TGN retention of KIT^mut^ in GIST cells by investigating the relationship between the PLCγ2–PKD2 pathway and PLDs using PLD inhibitors, knockdown experiments, immunofluorescence confocal microscopic analysis, and biochemical assays.

## Results

### KIT^mut^ is retained in the golgi/tgn region depending on PLD activity in GIST cells

**Fig. 1:**
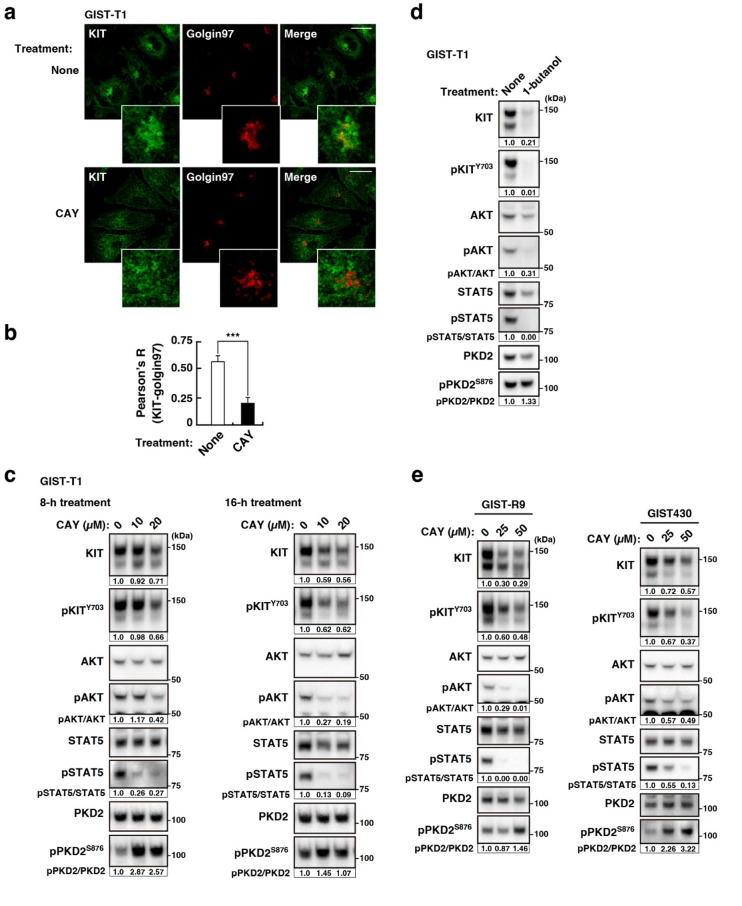
KIT^mut^ is trapped in the Golgi/TGN area in a manner dependent on PLD activity in GIST cells. (**a**) GIST-T1 cells were treated with 20 µM CAY10594 (CAY, a PLD inhibitor) for 4 h, then immunostained for KIT (green) and golgin97 (a Golgi/TGN marker, red). Insets show magnified images of the Golgi/TGN area. Scale bars, 20 μm. Note that KIT disappeared from the Golgi/TGN area in the presence of CAY10594. (**b**) The graph shows the correlation coefficients (Pearson’s R) between KIT and golgin97. Values represent mean ± SD (*n* = 16 each). A representative analysis from two independent experiments is shown. ***P < 0.001, Student’s *t*-test. (**c**) GIST-T1 cells were treated with CAY10594 for the indicated periods and then immunoblotted. pKIT^Y703^, phospho-KIT Y703; pPKD2^S876^, phospho-PKD2 S876. Relative band intensities of the upper band of KIT and pKIT^Y703^ normalised with each control sample are shown. Levels of pAKT, pSTAT5, and pPKD2^S876^ are expressed relative to the control cell sample, after normalisation with respective total protein levels. Note that the release of KIT^mut^ from the Golgi/TGN area by CAY10594 treatment inactivated AKT and STAT5 without decreasing pPKD2^S876^. (**d**) GIST-T1 cells were treated with 1% 1-butanol (inhibits PLD activity) for 8 h and then immunoblotted. (**e**) Imatinib-resistant cell lines, GIST-R9 cells (left) and GIST430 cells (right), were treated with CAY10594 for 8 h and then immunoblotted.

Previous studies have shown that PLD and PI4KIIIβ are activated downstream of PKD^18–20^. We hypothesised that PLD activity is also involved in the retention of KIT^mut^ in the Golgi/TGN region of GIST cells. To evaluate this hypothesis, we treated GIST-T1 cells, which endogenously express a constitutively active KIT^Δ560–578^ mutant (imatinib-sensitive)^31^with CAY10594 (an inhibitor of PLD activity)^32,33^ and examined whether PLD inhibition mimicked the inhibition of PKD2 on KIT localisation and growth signalling. First, we performed an immunofluorescence confocal microscopic assay with anti-KIT and anti-golgin97 (a Golgi/TGN marker) antibodies in GIST-T1 cells treated with 20 µM CAY10594 for 4 h. The cell shape became round after treatment for extended periods; thus, immunofluorescence data could be obtained from cells treated with the compound for a maximum of 4 h. As shown in Fig. 1a, KIT was found in the Golgi/TGN area together with golgin97 under normal conditions (upper panels), whereas it was absent in the CAY10594-treated cells (lower panels). We calculated the Pearson’s correlation coefficients (Pearson’s R) between KIT and golgin97 and confirmed that KIT in the Golgi/TGN region was significantly reduced by CAY10594 treatment (Fig. 1b, *P* < 0.001). This effect of CAY10594 was similar to that of the PKD inhibitor, CRT0066101, in releasing KIT from the Golgi/TGN region (compare Fig. 1a with Suppl. Fig. S1a). In immunoblotting, KIT was detected as a doublet band; the lower band is an immaturely glycosylated form, which is formed soon after synthesis, and the upper band is a complex-glycosylated form after reaching the *medial-*Golgi cisternae^12^. CAY10594 treatment decreased the KIT protein levels in a time- and dose-dependent manner (Fig. 1c, upper panels). The effect of the drug on phospho-KIT Y703 (pKIT^Y703^), a sign of KIT activation^1^correlated with that on KIT itself. This effect was similar to that of PKD2 inhibition by CRT0066101 and small interfering RNA (siRNA), which induced the release of KIT from the Golgi/TGN region and its subsequent degradation in lysosomes^16^. The reduction in the KIT band intensity indicated KIT release from the Golgi/TGN region. The CAY10594 effect was observed in the upper band of KIT, rather than in the lower band. Under PLD-inhibited conditions, the KIT downstream proteins, AKT and STAT5, were dephosphorylated and inactivated (Fig. 1c). Treatment with other PLD inhibitors, such as 1-butanol^21,34,35^yielded similar results (Fig. 1d) but differed in reducing the lower KIT band from CAY10594. At present, we cannot explain why CAY10594 barely affected the lower KIT band; therefore, we quantified the upper band throughout this study. Collectively, these results suggest that PLD activity is required for KIT^mut^ signalling by trapping the mutant in the Golgi/TGN region. Importantly, neither CAY10594 nor 1-butanol decreased phospho-PKD2 S876 (pPKD2^S876^) levels; however, both treatments transiently enhanced pPKD2^S876^ levels (Fig. 1c, d). These results indicate that PKD2 is not a downstream target of PLD2. We previously reported that not all PKD2 is activated by KIT in GIST cells^16^suggesting that an upstream molecule for PKD2, other than KIT, may be activated in the presence of CAY10594. We assumed that KIT-independent PKD2 activation does not participate in KIT retention. Next, we examined the effect of PLD inhibition with CAY10594 in imatinib-resistant GIST cell lines, GIST-R9 (*KIT*^*Δ560–578,D820V*^) and GIST430 cells (*KIT*^*Δ560–576,V654A*^). Although higher concentrations of the compound were required, GIST-R9 and GIST430 cells showed results similar to those of CAY10594-treated GIST-T1 cells; the protein levels of KIT decreased, AKT and STAT5 were dephosphorylated, and pPKD2^S876^ was not reduced (Fig. 1e; Suppl. Fig. S1b). Taken together, these results suggest that PLD, a presumed PKD effector, plays a key role in Golgi/TGN retention of KIT^mut^.

### KIT^mut^ released from the golgi/tgn migrates to lysosomes in GIST cells

**Fig. 2:**
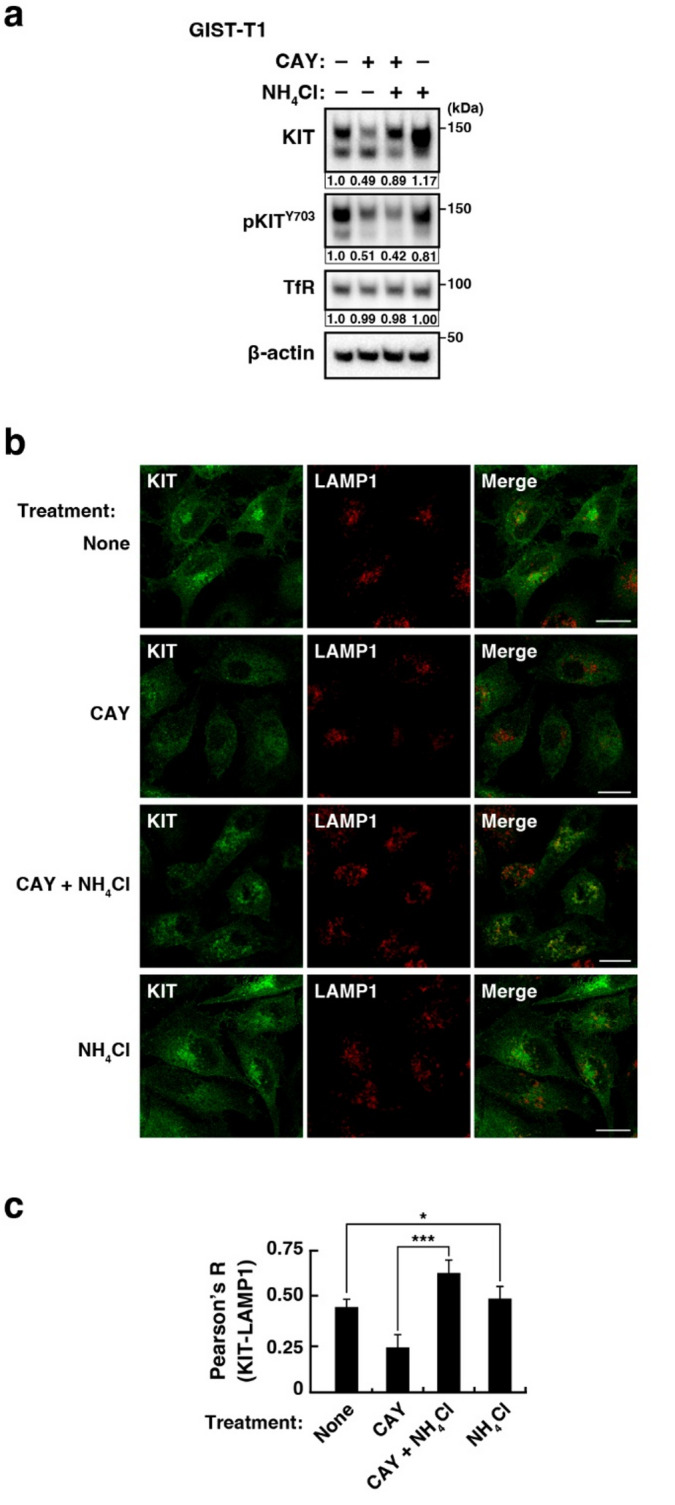
CAY10594-induced reduction of KIT is due to lysosomal degradation. (**a**, **b**) GIST-T1 cells were treated with 20 µM CAY10594 (CAY, a PLD inhibitor) and/or 20 mM NH_4_Cl (inhibits lysosomal proteases) for (**a**) 16 h or (**b**) 4 h. (**a**) Lysates were immunoblotted for KIT, phospho-KIT Y703 (pKIT^Y703^), transferrin receptor (TfR, a recycling endosome protein), and β-actin. Relative band intensities normalised with each control sample are shown. The upper bands of KIT were quantified. (**b**) Cells were immunostained with KIT (green) and LAMP1 (lysosome-associated membrane protein 1, red). Scale bars, 20 μm. Note that KIT was found in lysosomes in GIST cells treated with CAY10594 plus NH_4_Cl. (**c**) The graph shows the correlation coefficients (Pearson’s R) between KIT and LAMP1. Values represent mean ± SD (*n* = 15–29). A representative analysis from two independent experiments is shown. *P < 0.05, ***P < 0.001, Student’s *t*-test.

Recently, we reported that in PKD2-inhibited GIST cells, KIT^mut^ is released from the Golgi/TGN region into the PM and is subsequently degraded in lysosomes^16^. To examine whether the CAY10594-induced KIT reduction was lysosome-dependent, we treated GIST-T1 cells with CAY10594 and ammonium chloride (NH_4_Cl), which inhibits lysosomal proteases^36^followed by immunoblotting. Figure 2a shows that CAY10594 alone decreased the protein level of KIT, whereas the level of KIT was restored in cells treated with CAY10594 and NH_4_Cl. Levels of the transferrin receptor, which circulates between the PM and endosomes^37^were not affected by these treatments, suggesting that CAY10594 specifically acts on KIT^mut^ trafficking. Phospho-KIT Y703 (pKIT^Y703^) was not restored by NH_4_Cl treatment, indicating that KIT is incorporated into lysosomes after dephosphorylation. Previous studies showed that endocytosed RTK undergoes a “pit stop” on the ER to interact with protein tyrosine phosphatase (PTP) for its dephosphorylation^38,39^. KIT may also be dephosphorylated before its incorporation into lysosomes. Thus, pKIT levels did not increase in NH_4_Cl-treated cells. Immunofluorescence data showed that, in the presence of CAY10594 and NH_4_Cl, KIT was present in the lysosome-associated membrane protein 1-positive (LAMP1-positive) region (Fig. 2b, c). CAY10594, therefore, has an effect similar to that of PKD2 inhibition on the distribution of KIT^mut^ in GIST cells. These results suggest that KIT^mut^ is degraded in lysosomes after CAY10594-induced release from the Golgi/TGN region.

### PLD2 knockdown phenocopies the effect of CAY10594 treatment in GIST-T1 cells

**Fig. 3:**
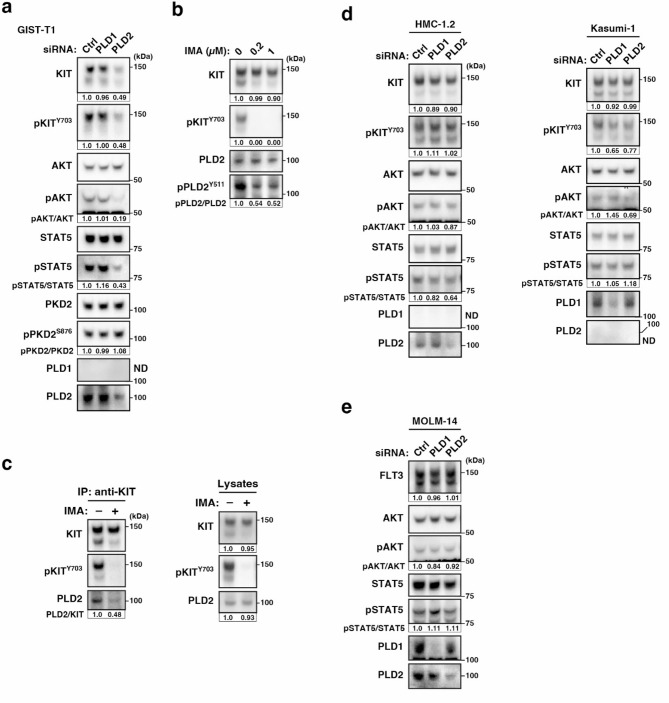
PLD2 is a downstream molecule of KIT^mut^ in GIST-T1 cells. (**a**) GIST-T1 cells were transfected with *PLD1*-targeted siRNA or *PLD2*-targeted siRNA and cultured for 30 h. Lysates were immunoblotted with the indicated antibodies. pKIT^Y703^, phospho-KIT Y703; pPKD2^S876^, phospho-PKD2 S876. ND, not detected. Relative band intensities of the upper band of KIT and pKIT^Y703^ normalised with each control sample are shown. Levels of pAKT, pSTAT5, and pPKD2^S876^ are expressed relative to the control cell sample, after normalisation with respective total protein levels. (**b**, **c**) GIST-T1 cells were treated with imatinib (IMA, a KIT tyrosine kinase inhibitor) for 8 h. (**b**) Lysates were immunoblotted. pPLD2^Y511^, phospho-PLD2 Y511. (**c**) KIT in cells treated with 200 nM imatinib was immunoprecipitated. The immunoprecipitates were immunoblotted. IP, immunoprecipitation. Immunoblots of a control antibody IP are shown in Suppl. Fig. S2c. (**d**, **e**) HMC-1.2 (MCL, *KIT*^*D816V*^), Kasumi-1 (AML, *KIT*^*N822K*^), or MOLM-14 cells (AML, *FLT3*^*ITD*^) were transfected with PLD1-targeted siRNA or PLD2-targeted siRNA and cultured for 48 h. Lysates were immunoblotted with the indicated antibodies. ND, not detected.

Mammalian PLDs comprise six structurally related proteins: PLD1, PLD2, PLD3, PLD4, PLD5, and PLD6^30,40^. PLD1 and PLD2 have PA-producing PLD activity not only in the PM but also in the Golgi/TGN, whereas no canonical PLD activity of PLD3, PLD4, or PLD5 has been reported^30^. PLD6 hydrolyses cardiolipin to generate PA in the mitochondria, a process called mito-PLD. Therefore, we examined whether knockdown of PLD1 or PLD2 phenocopied the effect of chemical inhibition of PLD in GIST. In PLD2-knockdown GIST-T1 cells, KIT reduction, a sign of release from the Golgi/TGN region, and suppression of KIT signalling were observed (Fig. 3a). Consistent with the immunoblotting data, KIT was reduced by PLD2 knockdown, whereas it was not affected by control or PLD1 siRNA (Suppl. Fig. S2a). Similar to the CAY10594 treatment, PLD2 knockdown did not decrease the expression of pPKD2^S876^. PLD1 protein expression was not detected in this cell line; thus, PLD1 siRNA did not affect KIT retention or growth signalling (Fig. 3a and Suppl. Fig. S2a). Therefore, PLD2 plays a pivotal role in Golgi/TGN retention of KIT in GIST-T1 cells. At this stage, we could not determine whether PLD1 was important for KIT retention. Next, we investigated whether PLD2 was downstream of KIT^mut^. As shown in Fig. 3b, imatinib, a KIT tyrosine kinase inhibitor, markedly decreased the phosphorylation of PLD2 at the residue Y511 (pPLD2^Y511^), which is a sign of PLD2 activation. At 0.2 µM the drug completely inhibited KIT activity, and the upper band remained, compared with that in CAY10594-treated cells. In imatinib-treated GIST cells, after reaching the PM, KIT is retained because its internalisation toward lysosomes is dependent on tyrosine kinase activity^12,15,16^. Thus, KIT levels remain despite decreasing pPLD2^Y511^. Because the new synthesis of KIT depends on its kinase activity^40^the lower band, which is reflected soon after synthesis, is decreased by imatinib. Furthermore, we performed coimmunoprecipitation assays to examine the association of KIT with PLD2. We used an anti-ALK antibody as a control because GIST-T1 cells did not express ALK (Suppl. Fig. S2b). PLD2 co-immunoprecipitated with KIT compared to the control, and this interaction was suppressed by imatinib treatment (Fig. 3c and Suppl. Fig. S2b, c). These results suggest that PLD2 is located downstream of KIT^mut^ in GIST-T1 cells.

Next, we examined the subcellular localisation of PLD2. In our immunofluorescence assay, we were unable to detect endogenous PLD2, probably owing to the low ability of the antibodies. Transiently transfected MYC-tagged PLD2 was present in the cytosolic and endomembrane fractions containing Golgi/TGN (Suppl. Fig. S2d, e). Considering that PLD2-MYC fluorescence was observed throughout GIST-T1 cells and was barely observed in the Golgi/TGN region, these results indicate that a small fraction of PLD2 is associated with KIT^mut^ in GIST cells.

Next, we examined whether PLD knockdown had a similar effect on RTK^mut^ expression in leukaemia cells as on KIT^mut^ expression in GIST-T1 cells. We have previously reported that KIT^mut^ in AML cells (Kasumi-1) and MCL cells (HMC-1.2) flows normally from the Golgi/TGN to the PM and immediately moves to the endosome–lysosome compartments^11,13,15^. We were unable to determine the expression of PLD2 and PLD1 in Kasumi-1 and HMC-1.2 cells, respectively. As shown in Fig. 3d, the effect of PLD knockdown on KIT protein levels in Kasumi-1 and HMC-1.2 was less than that in GIST-T1 cells. In addition, we recently showed that, in AML cells, the constitutively active FLT3 internal tandem duplication (FLT3-ITD) mutant is retained in the Golgi/TGN in a manner dependent on its tyrosine kinase activity. It activates AKT and STAT5 in the Golgi/TGN and ER, respectively^41,42^. We examined whether PLD1/2 knockdown in FLT3-mutated cells was similar to that observed in GIST-T1 cells. The knockdown of PLD1 or PLD2 did not affect the protein levels of FLT3 or AKT activation in the AML cell line MOLM-14 (Fig. 3e), indicating that FLT3-ITD is intracellularly retained in a manner independent of PLD2. Furthermore, FLT3-ITD-dependent STAT5 activation in the ER was unaffected in PLD-knockdown cells. This supports our findings that Golgi/TGN retention in the FLT3-ITD occurs in a PKD2-independent manner^16^. Collectively, these results suggest that PLD activity is specifically involved in Golgi/TGN retention of KIT^mut^ in GIST cells.

### KIT–PLCγ2–PKD2 separately activates PI4KIIIβ and PLD2

**Fig. 4:**
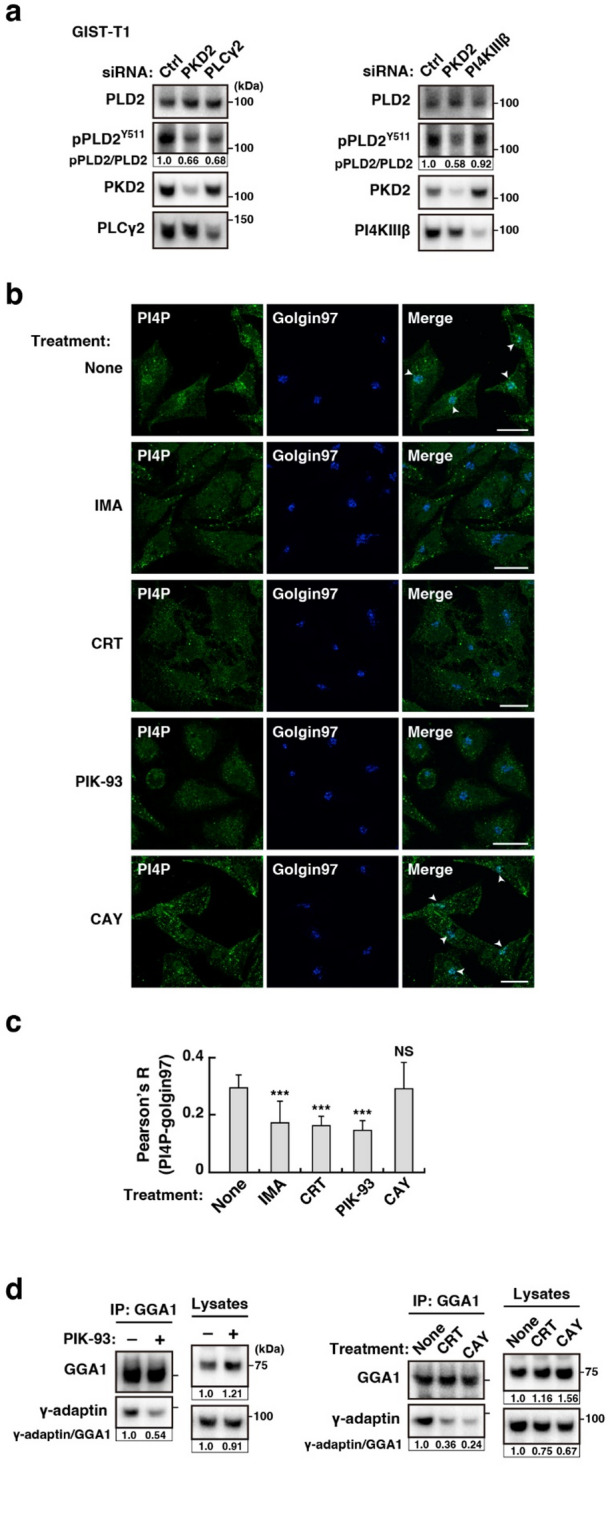
In GIST-T1 cells, PKD2, but not PI4KIIIβ, is an upstream molecule of PLD2. (**a**) GIST-T1 cells were transfected with the indicated siRNAs and cultured for 30 h. Lysates were immunoblotted with the indicated antibodies. pPLD2^Y511^, phospho-PLD2 Y511. Levels of pPLD2^Y511^ are expressed relative to the control cell sample after normalisation with PLD2. (**b**) GIST-T1 cells were treated with 200 nM imatinib (IMA, a KIT tyrosine kinase inhibitor), 10 µM CRT0066101 (CRT, a PKD inhibitor), 20 µM PIK-93 (a PI4KIIIβ inhibitor), or 20 µM CAY10594 (CAY, a PLD inhibitor) for 4 h. Cells were immunostained with anti-PI4P (green) and golgin97 (a Golgi/TGN marker, blue). Arrowheads indicate the Golgi/TGN area. Scale bars, 20 μm. (**c**) The graph shows the correlation coefficients (Pearson’s R) between PI4P and golgin97. Values represent mean ± SD (*n* = 11–22). Data were subjected to one-way analysis of variance (ANOVA) with Dunnett’s multiple comparison *post-hoc* test. A representative analysis from two independent experiments is shown. ***P < 0.001, NS, not significant. Note that PI4P in the Golgi/TGN area was decreased by treatment with imatinib, CRT0066101, and PIK-93, whereas it was retained in the presence of CAY10594. (**d**) GIST-T1 cells were treated with 20 µM PIK-93, 10 µM CRT0066101, or 20 µM CAY10594 for 8 h. GGA1 was immunoprecipitated with anti-GGA1 antibody. The immunoprecipitates were immunoblotted for GGA1 and γ-adaptin. IP, immunoprecipitation. Immunoblots of a control antibody IP are shown in Suppl. Fig. S2f.

**Fig. 5:**
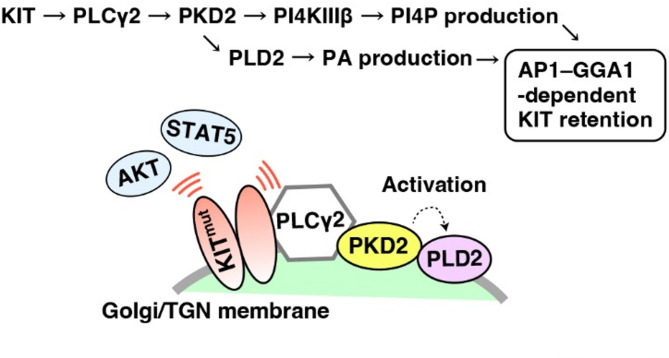
Model of PLD2-dependent Golgi retention of KIT mutant in GIST cells. PKD2 separately activates PLD and PI4KIIIβ in GIST cells. Both enzymatic products, PI4P and phosphatidic acid (PA), play direct or indirect roles in the association of GGA1 with AP1, which is important for Golgi/TGN retention of KIT^mut^. If these pathways are blocked, KIT^mut^ is released from the Golgi/TGN region, resulting in its lysosomal degradation.

Finally, we asked where PLD2 is located in the KIT–PLCγ2–PKD2–PI4KIIIβ–AP1–GGA1 pathway. Figure 4a shows that the knockdown of PLCγ2 as well as PKD2 reduced the pPLD2^Y511^, supporting previous reports that PLDs are downstream molecules of PKD^18–20^. On the other hand, pPLD2^Y511^ was maintained in PI4KIIIβ-knocked down cells compared with PKD2-knocked down cells, indicating that PLD2 is not a downstream molecule of PI4KIIIβ. Next, we examined the PI4P levels in the Golgi/TGN region. Imatinib (KIT inhibitor), CRT0066101 (PKD inhibitor), and PIK-93 (PI4KIIIβ inhibitor) significantly decreased PI4P in the Golgi/TGN region (Fig. 4b, c; *P* < 0.001), consistent with our previous report. In contrast, PI4P was found in the Golgi/TGN area in the presence of the PLD inhibitor CAY10594 (Fig. 4b, c). Our recent study showed that activation of the KIT–PLCγ2–PI4KIIIβ pathway is a cause of the association of γ-adaptin and GGA1. Inhibition of PI4KIIIβ, PKD2, and PLD2 decreased γ-adaptin interaction with GGA1 (Fig. 4d and Suppl. Fig. S2f). Collectively, these results suggest that PKD2 separately activates PLD2 and PI4KIIIβ to recruit AP1 and GGA1 (Fig. 5). Both PI4KIIIβ-produced PI4P and PLD2-produced PA probably contribute to the association of AP1 with GGA1. Although both AP1 and GGA1 play a role in protein trafficking from the TGN^17^we hypothesised that aberrantly, probably excessively, recruited AP1 and GGA1 disrupt the normal trafficking of KIT.

We found that the imatinib-resistant GIST cell lines, GIST-R9 and GIST430, expressed PLD1, unlike GIST-T1 cells. Quantification results indicated that in GIST-R9 and GIST430 cells, PLD2 knockdown reduced the protein levels of KIT (to 74% in GIST-R9 and 81% in GIST430), resulting in a decrease in pKIT^Y703^ (to 64% in GIST-R9 and 78% in GIST430) (Suppl. Fig. S3a). In GIST430 cells, the knockdown of PLD1 decreased KIT by 16%, indicating that the involvement of PLD1 in KIT retention depends on the cellular context. The changes in phospho-AKT (pAKT) and pSTAT5 levels did not correlate with a reduction in KIT or pKIT. Furthermore, the addition of PLD2 siRNA to PLD1 siRNA did not have an additive effect on KIT reduction (Suppl. Fig. S3b). Because the effect of PLD2 knockdown on KIT levels in GIST-R9 and GIST430 cells was less than that in GIST-T1 cells, these imatinib-resistant GIST cells may compensate for the loss of PLD1 and PLD2 proteins and restore PA levels in PLD1/PLD2-knocked cells. In addition, dephosphorylation of AKT and STAT5 may be impaired in the PLD2-knocked down imatinib-resistant cells. At present, we cannot explain the reason for this. Further studies are required to elucidate these compensatory mechanisms.

## Discussion

In this study, we demonstrated that PLD activity was required for Golgi/TGN retention of KIT^mut^ in GIST (Fig. 5). The PLD inhibitors CAY10594 and 1-butanol moved KIT^mut^ from the Golgi/TGN, resulting in downstream inactivation. After release from the Golgi/TGN region, KIT^mut^ is incorporated into lysosomes for degradation. PLD inhibitors have a similar effect as PKD inhibitors on KIT^mut^ localisation and growth signalling in GIST cells. KIT^mut^ activates PLD2 via the PLCγ2–PKD2 pathway. Activation of PLD2 by KIT^mut^ leads to the mutant retention in the Golgi/TGN, suggesting that KIT^mut^ is retained via a positive feedback loop. PKD2 could separately activate PLD2 and PI4KIIIβ for producing PA and PI4P, respectively. Both PLD2 and PI4KIIIβ are required for the association of AP1 with GGA1, which is a cause of Golgi retention of KIT^mut^ in GIST cells. The loss-of-function of PLD does not affect the signalling of other RTK^mut^ mutations, such as FLT3-ITD, in AML. These findings suggest that KIT^mut^ separately activates PLD2 and PI4KIIIβ through the PLCγ2–PKD2 pathway for its Golgi retention in GIST cells.

Previous studies have demonstrated Golgi/TGN retention by RTKs other than KIT^mut^ and FLT3-ITD. Constitutively active forms of PDGFRA, FGFR3, and TRKA are found in the Golgi/TGN region and are autophosphorylated in cancer cells^43–47^. In hepatocellular carcinoma cells, overexpressed MET is retained in the Golgi/TGN region in a manner dependent on its tyrosine kinase activity^48^. Tyrosine phosphorylates HER3 kinase, leading to the autonomous proliferation of host cells. Non-receptor-type oncogenic proteins, such as Src-family tyrosine kinases, RAS GTPases, and the mammalian target of rapamycin, have been suggested to use the Golgi/TGN membrane as their signalling platform^49–54^. PLD is located downstream of RAS^55,56^. Investigating whether RAS uses PLD for Golgi/TGN localisation is of interest. Therefore, it is of substantial interest to determine whether PLD plays a role in the Golgi/TGN retention of oncogenic signal transduction proteins other than GIST KIT^mut^.

PLD1 and PLD2 generate PA and choline from PC located in the lipid bilayer^57^. PA plays multiple roles in various cellular processes including lipid biogenesis, membrane trafficking, and cytoskeletal reorganisation, and acts as a scaffold for signal transduction^58–60^. In the transport carrier formation process at the Golgi/TGN membrane, PA provides platforms for membrane-trafficking proteins and creates membrane curvature owing to its cone-like shape^27–29^. In GIST cells, autophosphorylation of KIT in the Golgi/TGN membrane, which does not occur in normal cells, aberrantly activates PLD2 via the PLCγ2–PKD2 pathway, probably resulting in local dysregulated PA production. We hypothesised that abnormal local PA production would stop the export of KIT^mut^ from the Golgi/TGN region. Abnormally generated PA may play a role in the induction of the AP1–GGA1 interaction, which is involved in KIT retention. GGA1 and AP1 carry cargoes from the TGN^17^; thus, the above statements may be counterintuitive. We assumed that aberrantly, probably excessively, recruited adaptor proteins disrupted the normal trafficking of KIT. Analysis of PA distribution using fluorescence imaging and quantification of PA amounts will help us understand the precise mechanism of PA-dependent Golgi/TGN retention by KIT^mut^ in GIST cells.

In the present study, we identified PLD2 as a downstream target of KIT^mut^ in GIST. In mast cells, SCF-stimulated normal KIT at the PM induces PLD activation, resulting in chemical mediator release^61,62^. The SCF–KIT axis physiologically activates PLD in haematopoietic cells, melanocytes, germ cells in the testes, and interstitial Cajal cells. Therefore, examining the physiological role of PLD in these cells is of great interest.

Previous studies reported that ADP-ribosylation factor (ARF) proteins act as mediators between PKD and PLD^18–20,63,64^. In our recent report, we knocked down Golgi-localised ARFs such as ARF1, ARF4, and ARF5 in GIST-T1 cells^65^. However, the knockdown of ARF1, ARF4, or ARF5 did not phenocopy PLD2 knockdown. Furthermore, since the simultaneous knockdown of ARF1, ARF4, and ARF5 blocks the ER export of KIT^mut^, a conclusion cannot be drawn from a simple ARF loss-of-function study. Cell-free system methods and protein-protein interaction analyses will be powerful tools for understanding the precise mechanism of PLD activation through ARF function.

In imatinib-resistant GIST cell lines, knockdown of PLD1 and PLD2 could not fully explain the effect of the PLD inhibitor CAY10594. Genetic alterations occur not only in the *KIT* gene^66^. Multiple enzymes such as diacylglycerol kinases, lysophosphatidic acid acyltransferases, PA phosphatases, and phospholipase A are involved in controlling intracellular PA levels^29,67^. In GIST-R9 and GIST430 cells, alterations in the expression of PA-related proteins may compensate for the siRNA-induced loss of PLD1 and PLD2. CAY10594 may have off-target effects, particularly in imatinib-resistant GIST cell lines. Further studies are required to understand the mechanisms underlying Golgi retention induced by KIT^mut^ in imatinib-resistant cells.

In conclusion, we showed that in addition to PI4KIIIβ, PLD2 is an effector protein of the KIT–PLCγ2–PKD2 cascade, and its enzymatic product PA may be a key player for KIT retention in the Golgi/TGN in GIST cells. PLD2 maintains the uncontrolled growth signalling of KIT^mut^ by trapping the mutant in the Golgi/TGN. Our findings provide novel insights into the role of PLD2 in GIST growth. Moreover, from a clinical perspective, our findings offer a new strategy for GIST treatment by releasing KIT^mut^ from the signalling platform.

## Materials and methods

### Cell culture

GIST-T1 (Cosmo Bio, Tokyo, Japan) and GIST-R9 cells were cultured at 37 °C in Dulbecco’s modified Eagle’s medium (DMEM) supplemented with 10% fetal calf serum (FCS), penicillin, and streptomycin (Pen/Strep). An imatinib-resistant GIST cell line, GIST-R9, was generated from GIST-T1 cells^66^. GIST-R9 cells were cultured with 1 µM imatinib as described previously^66^. GIST430/654 (hereafter referred to as GIST430) cells were kindly provided by Dr. Jonathan Fletcher (Dana-Farber Cancer Institute, Boston, MA, USA). GIST430 cells were cultured at 37 °C in Iscove’s modified Dulbecco’s medium (IMDM) supplemented with 15% FCS and Pen/Strep. GIST430 cells were maintained in the presence of 100 nM imatinib^68^. Kasumi-1, NB-1 (JCRB Cell Bank, Osaka, Japan), MOLM-14 (DSMZ, Braunschweig, Germany), and HMC-1.2 cells^69^ were cultured at 37 °C in RPMI1640 supplemented with 10% FCS and Pen/Strep. All human cell lines were authenticated by short tandem repeat analysis at the JCRB Cell Bank and tested for *mycoplasma* contamination using the MycoAlert Mycoplasma Detection Kit (Lonza, Basel, Switzerland).

### Antibodies

The antibodies used for immunoblotting, immunoprecipitation, and immunofluorescence are listed in Supplementary Table S1.

### Chemicals

CAY10594, imatinib mesylate (Cayman Chemical, Ann Arbor, MI, USA), PIK-93, and CRT0066101 (Selleck Chemicals) were dissolved in dimethyl sulfoxide. NH_4_Cl and 1-butanol were purchased from Sigma-Aldrich (St. Louis, MO) and Fujifilm Wako Chemicals (Osaka, Japan), respectively.

### Gene silencing with SiRNA

For silencing, *PLD1*, *PLD2*, *PLCγ2*, *PKD2*, and *PI4KIIIβ* ON-TARGETplus SMARTpool siRNAs were purchased from Horizon Discovery (Waterbeach, UK). A list of the siRNAs used in this study is provided in Supplementary Table S2. Electroporation was performed using the NEON Transfection System (Thermo Fisher Scientific), according to the manufacturer’s instructions.

### Immunofluorescence confocal microscopy

GIST-T1 cells were cultured on poly L-lysine-coated coverslips and fixed with 4% paraformaldehyde (PFA) for 20 min at room temperature. The fixed cells were permeabilised and blocked for 30 min in Dulbecco’s phosphate-buffered saline (D-PBS(-)) supplemented with 0.1% saponin and 3% bovine serum albumin (BSA), and then incubated with primary and secondary antibodies for 1 h each. After washing with D-PBS(-), cells were mounted using Fluoromount (Diagnostic BioSystems, Pleasanton, CA, USA). Confocal images were obtained using a FLUOVIEW FV3000 (Olympus, Tokyo, Japan) or TCS SP8 (Leica, Wetzlar, Germany) laser scanning microscope. Composite figures were prepared using FV31S-SW (Olympus), Leica Application Suite X Software (Leica), Photoshop, and Illustrator (Adobe, San Jose, CA, USA). Pearson’s R was calculated using the ImageJ software (Fiji, version 1.0; National Institutes of Health (NIH), Bethesda, MD, USA).

### Immunostaining for PI4P visualisation

GIST-T1 cells were treated with 50 mM NH_4_Cl in D-PBS(-) before fixation with 2% PFA. Permeabilisation and staining were performed as previously described^16,70^. For PI4P staining, anti-PI4P mouse IgM (Echelon Biosciences, Salt Lake City, UT, USA) and goat Alexa Fluor 488-conjugated anti-mouse IgM (Thermo Fisher Scientific) were used as primary and secondary antibodies, respectively. After washing with D-PBS(-), stained cells were fixed with 2% PFA for 15 min, treated with 50 mM NH_4_Cl in D-PBS(-), and washed with double-distilled water.

### Western blotting

Lysates prepared in a sodium dodecyl sulfate-polyacrylamide gel electrophoresis (SDS-PAGE) sample buffer were subjected to SDS-PAGE and electrotransferred onto polyvinylidene fluoride membranes. Generally, we split the membranes at 70 kDa molecular weight into two pieces for lysate immunoblotting. Briefly, 5% skim milk in Tris-buffered saline containing Tween 20 (TBST) was used to dilute the antibodies. For immunoblotting with anti-pKIT Y703 and anti-pPLD2 Y511 antibodies, the antibodies were diluted with 3% BSA in TBST. Immunodetection was performed using the Immobilon Western Chemiluminescent HRP Substrate (Sigma-Aldrich). Sequential re-probing of the membranes was performed after complete removal of the antibodies with Restore PLUS Western Blot Stripping Buffer (Thermo Fisher Scientific) and/or inactivation of peroxidase by 0.1% sodium azide. The results were analysed using a ChemiDoc XRC + with Image Lab software (Bio-Rad, Hercules, CA, USA). Full-length blots are presented in Supplementary Figures S4 and S5.

### Immunoprecipitation

Lysates from drug-treated GIST-T1 cells were prepared in NP-40 lysis buffer supplemented with 50 mM HEPES (pH 7.4), 10% glycerol, 1% NP-40, 4 mM EDTA, 100 mM NaF, 1 mM Na_3_VO_4_, protease inhibitor cocktail, 2 mM β-glycerophosphate, 2 mM sodium pyrophosphate, and 1 mM phenylmethylsulfonyl fluoride. Immunoprecipitation was performed at 4 °C for 5 h using Dynabeads Protein G beads pre-coated with anti-KIT, anti-GGA1, or anti-ALK antibodies. The immunoprecipitates were dissolved in SDS-PAGE sample buffer and immunoblotted.

### PLD2-MYC transfection

A gene encoding the carboxy-terminal MYC-tagged PLD2 (PLD2-MYC), inserted into the pRP vector for expression in mammalian cells, was purchased from Vector Builder (Yokohama, Japan; vector ID: VB230228-1368ssm). Transient transfection of PLD2-MYC was performed using PEIMAX (Polysciences, Warrington, PA, USA) or JetOPTIMUS (Polyplus-Transfection; Illkirch, France). PLD2-MYC expression was detected by immunoblotting and immunofluorescence using an anti-MYC antibody (71D10).

### Quantification and statistical analysis

Differences between two or more groups were analysed using a two-tailed Student’s *t*-test or one-way analysis of variance, followed by Dunnett’s multiple comparison *post-hoc* test. All significant differences were indicated at a 5% probability level. Experiments were repeated twice unless otherwise stated. Data on each leukaemia cell line were obtained from single experiments; however, we obtained data from three independent cell lines to repeat the experiments on leukaemia cells.

## Supplementary Information

Below is the link to the electronic supplementary material.

Supplementary Material 1

## Data Availability

All datasets used and/or analysed in the current study are available from the corresponding author upon reasonable request.

## Abbreviations

AML: Acute myelogenous leukaemia
AP1: Adaptor protein-1 complex
ARF: ADP-ribosylation factor
BSA: Bovine serum albumin
CAY: CAY10594
CRT: CRT0066101
DMEM: Dulbecco’s modified Eagle’s medium
D-PBS(-): Dulbecco’s phosphate-buffered saline
siRNA: Small interfering RNA
ER: Endoplasmic reticulum
ERK: Extracellular signal-regulated kinase
FCS: Fetal calf serum
FGFR: Fibroblast growth factor receptor
FLT3-ITD: FMS-like tyrosine kinase 3-intenal tandem duplication
GGA: Golgi-associated,γ-adaptin ear-containing, ARF-binding protein
GIST: Gastrointestinal stromal tumour
IP: Immunoprecipitation
LAMP1: Lysosome-associated membrane protein 1
MCL: Mast cell leukaemia
mut: Mutant
NH_4_Cl: Ammonium chloride
PA: Phosphatidic acid
PC-PLD: Phosphatidylcholine-selective phospholipase D
PDGFR: Platelet-derived growth factor receptor
Pearson’s R: Pearson’s correlation coefficients
Pen/Strep: Penicillin and streptomycin
PFA: Paraformaldehyde
PI3K: Phosphatidylinositol-3 kinase
PI4KIIIβ: Phosphatidylinositol-4 kinase IIIβ
PI4P: Pphosphatidylinositol 4-phosphate
PKD: Protein kinase D
pKIT: Phospho-KIT
PLCγ: Phospholipase Cγ
PM: Plasma membrane
RTK: Receptor tyrosine kinase
SCF-R: Stem cell factor receptor
SDS-PAGE: Sodium dodecyl sulfate-polyacrylamide gel electrophoresis
siRNA: Small interfering RNA
STAT: Signal transducer and activator of transcription
TBST: Tris-buffered saline with Tween 20
TfR: Transferrin receptor
TGN: Trans-Golgi network
TKI: Tyrosine kinase inhibitor
TRKA: Tropomyosin receptor kinase A
