# Supplementary material for: Golgi retention of KIT in gastrointestinal stromal tumour cells is phospholipase D activity-dependent
Source: Sci Rep. 2025 Aug 6;15:28778. doi: 10.1038/s41598-025-14739-w (PMC12328827; doi:10.1038/s41598-025-14739-w)
Supplement: Supplementary file 1 — Supplementary Material 1 [file 41598_2025_14739_MOESM1_ESM.pdf]

Supplementary Information

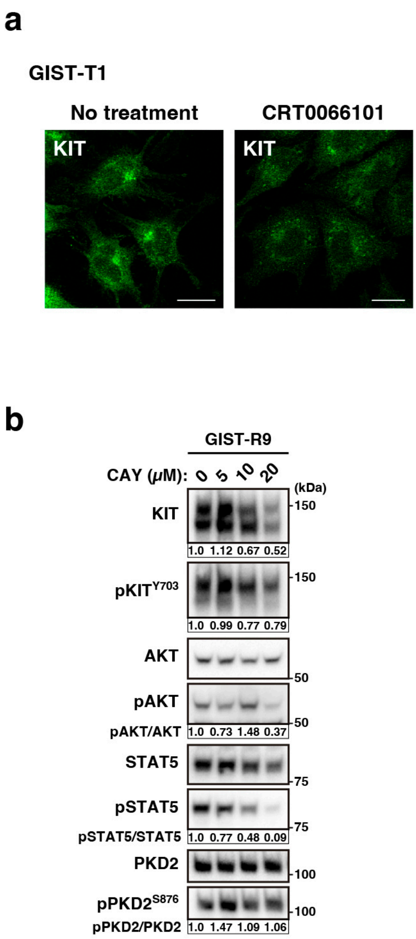

Supplementary Figure S1. KIT^{mut} is retained in the Golgi/TGN region in a PKD2-dependent manner in GIST-T1 cells.
(**a**) GIST-T1 cells were treated with 20 μM CRT0066101 (a PKD inhibitor) for 4 h and then immunostained with anti-KIT antibody. Scale bars, 20 μm. (**b**) GIST-R9 cells were treated with CAY10594 (CAY, a PLD inhibitor) for 8 h and then immunoblotted. pKIT^{Y703}, phospho-KIT Y703; pPKD2^{S876}, phospho-PKD2 S876. Relative band intensities of the upper band of KIT and pKIT^{Y703} normalised with each control sample are shown. Levels of pAKT, pSTAT5, and pPKD2^{S876} are expressed relative to the control cell sample, after normalisation with respective total protein levels.

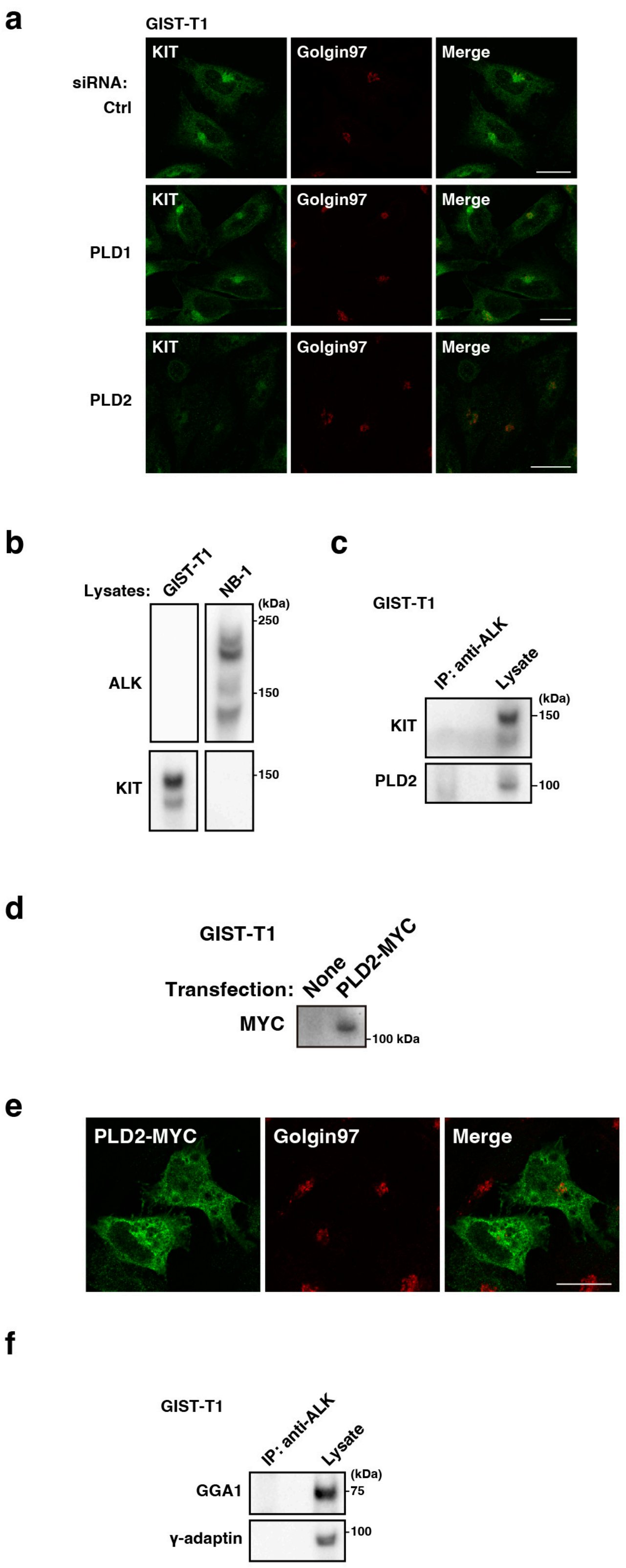

Supplementary Figure S2. Effect of PLD2 knockdown and subcellular localisation of PLD2 in GIST-T1 cells.

(**a**) GIST-T1 cells were transfected with ***PLD1***-targeted siRNA or ***PLD2***-targeted siRNA and cultured for 30 h, then immunostained for KIT (green) and golgin97 (Golgi marker, red). Scale bars, 20 μm. (**b**) Lysates from GIST-T1 and NB-1 (neuroblastoma) cell lines were immunoblotted with the indicated antibodies. GIST-T1 did not express ALK protein. (**c**) Neither KIT nor PLD2 was detected in the anti-ALK immunoprecipitates. (**d**,**e**) GIST-T1 cells were transfected with PLD2-MYC for 24 h. (**d**) Lysates were immunoblotted with an anti-MYC antibody. (**e**) Cells were immunostained with anti-MYC (green) and anti-golgin97 (red) antibodies. Scale bar, 20 μm. (**f**) Neither GGA1 nor γ-adaptin was detected in the anti-ALK immunoprecipitates.

a

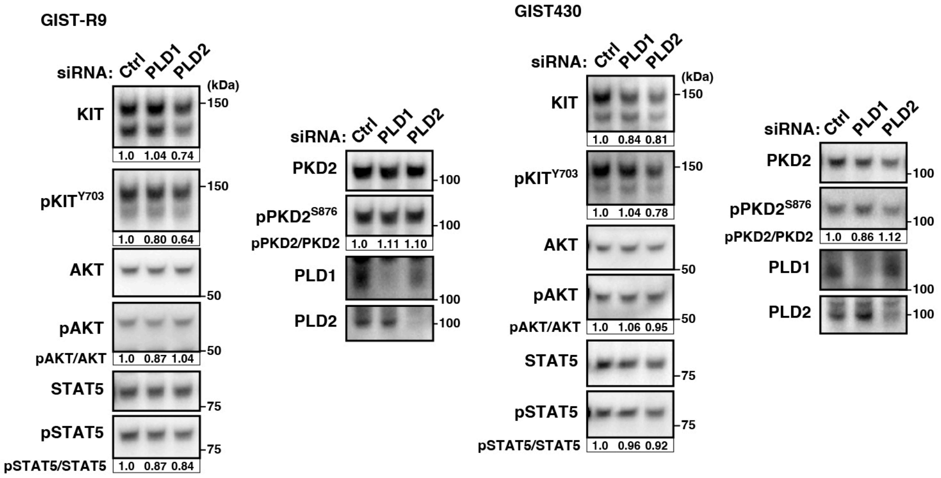

b

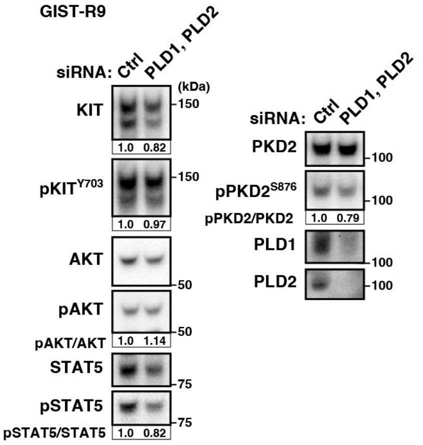

Supplementary Figure S3. Effect of PLD knockdown on GIST-R9 and GIST430 cells.

(**a**,**b**) GIST-R9 cells and GIST430 cells were transfected with the indicated siRNAs for 48 h. Lysates were immunoblotted with the indicated antibodies. pPKD2^{S876}, phospho-PKD2 S876. Relative band intensities of the upper band of KIT and pKIT^{Y703} normalised with each control sample are shown. Levels of pAKT, pSTAT5, and pPKD2^{S876} are expressed relative to the control cell sample after normalisation with respective total protein levels.

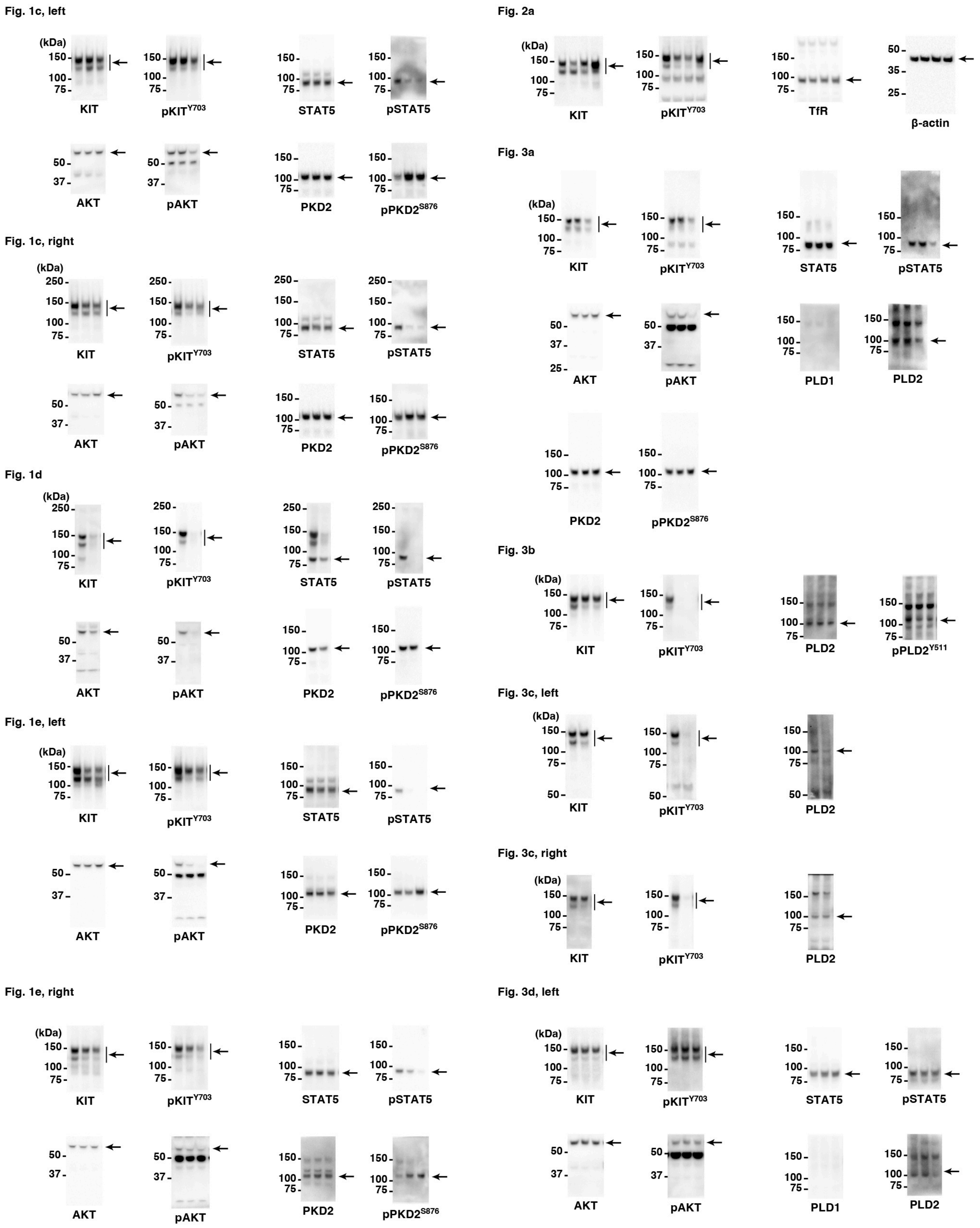

Supplementary Figure S4. Uncropped versions of the immunoblots of Figures 1c-3d.

Fig. 3d, right

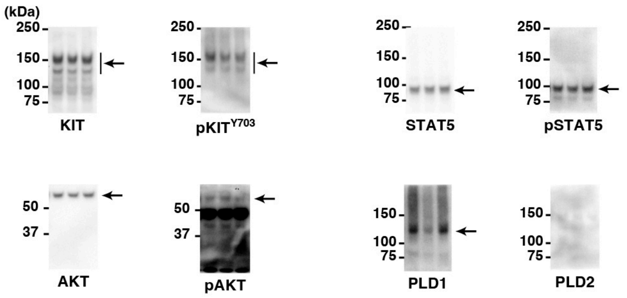

Fig. 3e

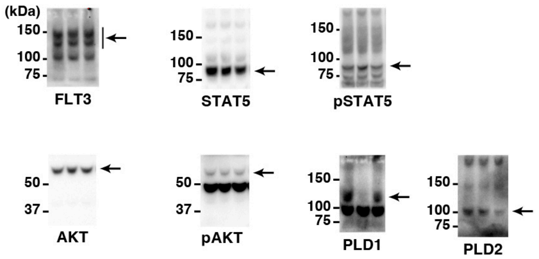

Fig. 4a, left

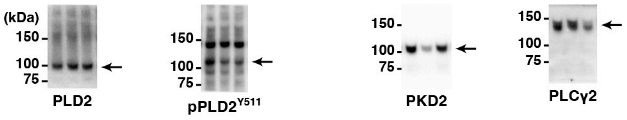

Fig. 4a, right

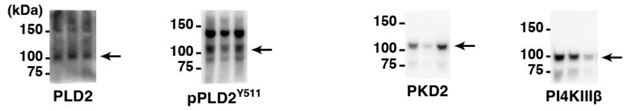

Fig. 4d, left

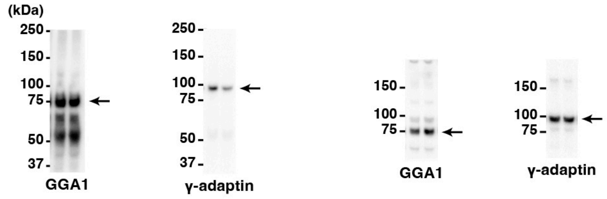

Fig. 4d, right

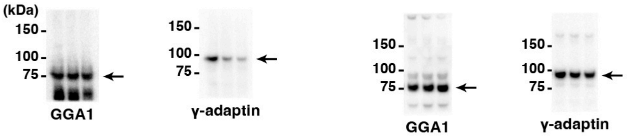

Supplementary Fig. S1b

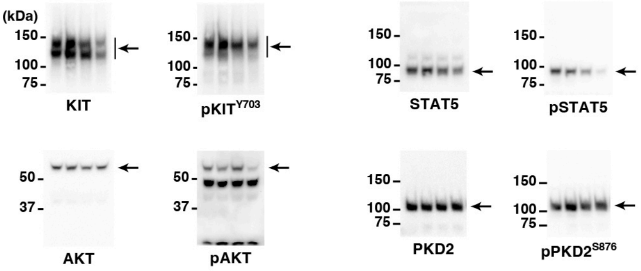

Supplementary Fig. S2b

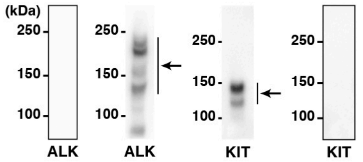

Supplementary Fig. S2c

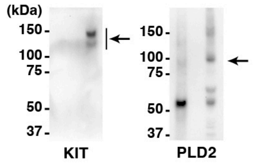

Supplementary Fig. S2d

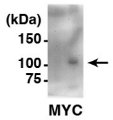

Supplementary Fig. S2f

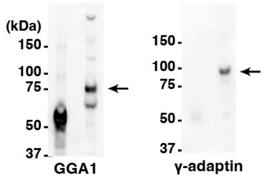

Supplementary Fig. S3a, left

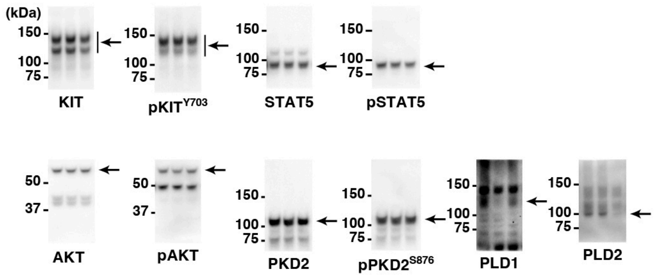

Supplementary Fig. S3a, right

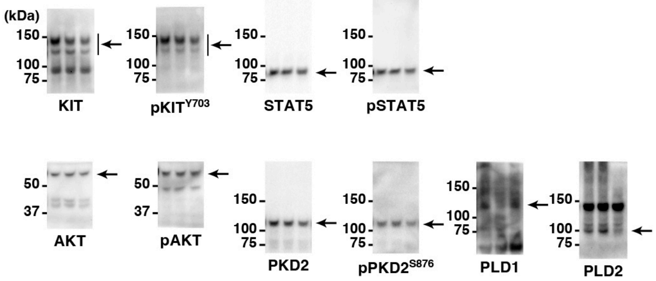

Supplementary Fig. S3b

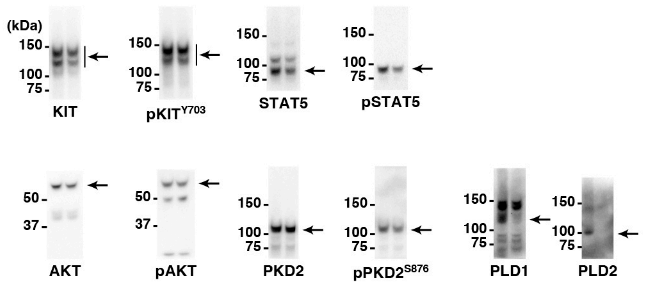

Supplementary Figure S5. Uncropped versions of the immunoblots of Figures 3d-4d and Supplementary Figures S1b-S3b.

Supplementary Table S1. List of antibodies

Antibody	Distribution source	Identifier
AKT (40D4) Mouse mAb	Cell Signaling Technology	Cat#2920; PRID: AB_1147620
AKT (clone 55) Mouse mAb	BD Transduction Laboratories	Cat#610861 RRID: AB_398180
Phospho-AKT (T308) (C31E5E) Rabbit mAb	Cell Signaling Technology	Cat#2965; PRID: AB_2255933
β-actin Rabbit polyclonal ab	Cell Signaling Technology	Cat#4967; PRID: AB_330288
ALK (D5F3) Rabbit mAb	Cell Signaling Technology	Cat#3633; PRID: AB_11127207
ALK Rabbit polyclonal ab	Abclonal	Cat#A0766; PRID: AB_2757389
FLT3 (8F2) Rabbit mAb	Cell Signaling Technology	Cat#4967; PRID: AB_330288
γ-adaptin (clone 88) Mouse mAb	BD Transduction Laboratories	Cat#610386; PRID: AB_397768
GGA1 Rabbit polyclonal ab	Proteintech	Cat#25674-1-AP; PRID: AB_2880188
Golgin-97 (D8P2K) Rabbit mAb	Cell Signaling Technology	Cat#13192; PRID: AB_2798144
Golgin-97 (CDF4) Mouse mAb	Thermo Fisher Scientific	Cat#14-9767-82; PRID: AB_2573010
KIT (D13A2) Rabbit mAb	Cell Signaling Technology	Cat#3074; PRID: AB_1147633
KIT (D3W6Y) Rabbit mAb	Cell Signaling Technology	Cat#37805; PRID: AB_2799120
KIT (E-1) Mouse mAb	Santa Cruz Biotechnology	Cat#sc-17806; PRID: AB_626875
Phospho-KIT (Y703) (D12E12) Rabbit mAb	Cell Signaling Technology	Cat#3073; PRID: AB_1147635
LAMP1 (D4O1S) Mouse mAb	Cell Signaling Technology	Cat#15665; PRID: AB_2798750
MYC-Tag (71D10) Rabbit mAb	Cell Signaling Technology	Cat#2278; PRID: AB_10828091
PI4KIIIβ (clone 7) Mouse mAb	BD Transduction Laboratories	Cat#sc-17806; PRID: AB_626875
PI4P Mouse mAb	Echelon Biosciences	Cat#Z-P004; PRID: AB_11127796
PKD2 (D1A7) Rabbit mAb	Cell Signaling Technology	Cat#8188; PRID: AB_10829368
PKD2 (O95G1) Mouse mAb	BioLegend	Cat#617302; PRID: AB_2810668
Phospho-PKD2 (S876) (EP1496Y) Rabbit mAb	Abcam	Cat#ab51251; PRID: AB_882060
PLCγ2 (E5U4T) Rabbit mAb	Cell Signaling Technology	Cat#55512; PRID: AB_2799488
PLD1 Goat polyclonal ab	R&D SYSTEMS	Cat#AF5615; RRID: AB_2163858
PLD1 (F-12) Mouse mAb	Santa Cruz Biotechnology	Cat#sc-28314; RRID: AB_677324
PLD1 Rabbit mAb	Abcam	Cat#ab68150
PLD2 Goat polyclonal ab	R&D SYSTEMS	Cat#AF10123
Phospho-PLD2 (Y511) Rabbit polyclonal ab	Thermo Fisher Scientific	Cat#PA5-105377; PRID: AB_2816805
STAT5 (C-17) Rabbit polyclonal ab	Santa Cruz Biotechnology	Cat#sc-835; PRID: AB_632446
STAT5 (clone 89) Mouse mAb	BD Transduction Laboratories	Cat#610192; PRID: AB_397590
STAT5 (D2O6Y) Rabbit mAb	Cell Signaling Technology	Cat#94205; PRID: AB_2737403
STAT5 [pY694] Rabbit mAb	Cell Signaling Technology	Cat#4322; PRID: AB_10544692
Transferrin Receptor (H68.4) Mouse mAb	Thermo Fisher Scientific	Cat#13-6800; PRID: AB_2533029
HRP donkey anti-mouse IgG	Jackson ImmunoResearch	Cat#715-035-151; PRID: AB_2340771
HRP donkey anti-rabbit IgG	Jackson ImmunoResearch	Cat#711-035-152; PRID: AB_10015282
HRP donkey anti-goat IgG	Jackson ImmunoResearch	Cat#705-035-147; PRID: AB_2313587
Donkey anti-Rabbit IgG (H+L), Alexa Fluor 488	Thermo Fisher Scientific	Cat#A-21206; PRID: AB_2535792
Donkey anti-Mouse IgG (H+L), Alexa Fluor 568	Thermo Fisher Scientific	Cat#A-10037; PRID: AB_2534013
Donkey anti-Rabbit IgG (H+L), Alexa Fluor 647	Thermo Fisher Scientific	Cat#A-31573; PRID: AB_2536183
Goat anti-Mouse IgM (Heavy chain), Alexa Fluor 488	Thermo Fisher Scientific	Cat#A-21042; PRID: AB_2535711

Supplementary Table S2. List of siRNAs

Oligonucleotides	Distribution source	Cat#
ON-TARGETplus Human PKD2 (25865) siRNA - SMARTpool	Horizon Discovery	L-004197-00
ON-TARGETplus Human PLCγ2 (5336) siRNA - SMARTpool	Horizon Discovery	L-008339-02
ON-TARGETplus Human PI4KIIIβ (5298) siRNA - SMARTpool	Horizon Discovery	L-006777-00
ON-TARGETplus Human PLD1 (5337) siRNA – SMARTpool	Horizon Discovery	L-009413-00
ON-TARGETplus Human PLD2 (5338) siRNA – SMARTpool	Horizon Discovery	L-005064-00
ON-TARGETplus Non-targeting Pool	Horizon Discovery	D-001810-10-20
